# Assessment of Maternal Dietary Intake, Physical Activity Status, and Body Composition During Pregnancy: A Cross-Sectional Study

**DOI:** 10.3390/nursrep15030099

**Published:** 2025-03-14

**Authors:** Vasiliki Michou, Arsenios Tsiotsias, Panagiotis Eskitzis

**Affiliations:** Department of Midwifery, School of Healthcare Sciences, University of Western Macedonia, Keptse, 50200 Ptolemaida, Greece; atsiotsias@uowm.gr (A.T.); peskitzis@uowm.gr (P.E.)

**Keywords:** pregnancy, physical activity, nutrition questionnaire, bioelectrical impedance analysis

## Abstract

**Objective:** This study aimed to estimate the Greek population’s maternal dietary intake, physical activity status, and body composition during pregnancy. **Research method:** Forty-nine women during pregnancy, with a mean age of 31.08 ± 4.90 years old, were asked to fill in the Pregnancy Physical Activity Questionnaire (PPAQ) to assess their physical activity levels, the Food Frequency Questionnaire (FFQ) to assess the maternal dietary intake, and a Bioelectrical Impedance Analysis (BIA) to evaluate different body composition indices. **Results:** Variance analysis showed that the pregnancy trimester’s effect on various indices of BIA was statistically significant. Moreover, the results showed that pregnant women consume a median of 2135 kcal and 2012.10 mL of water per day, regardless of their trimester. The Pearson correlation analysis unveiled a significant positive correlation between energy (r = 0.795, *p* < 0.001), water (r = 0.759, *p* < 0.001), fat (r = 0.535, *p* = 0.029), and dietary fibers (r = 0.310, *p* < 0.001) with pregnancy trimester. According to the PPAQ in women, categorized based on their pregnancy trimester, the results showed that women in the third trimester were statistically more sedentary than those in the second and first trimesters, respectively. Lastly, multiple regression analysis showed that pregnancy trimester (*p* = 0.005), employment status (*p* = 0.040), economic status (*p* = 0.037), and higher BMI (*p* = 0.013), BFMI (*p* = 0.017), and FFMI (*p* = 0.024) values had a significant contribution to the model. **Conclusions:** Pregnancy trimester has a significant impact on different indices of BIA and nutrients based on the FFQ, while physical activity levels decrease dramatically during pregnancy.

## 1. Introduction

The reproductive period is crucial in an individual’s life because it allows for the identification of variables associated with the risk of developing chronic diseases or future complications in offspring. The nutritional profile during this developmental period is significant, as it is considered a determining factor for the risk of chronic diseases throughout life and is potentially a modifiable risk factor [1]. The World Health Organization (WHO) has established dietary guidelines for pregnant women, but clear recommendations for women’s nutritional needs from conception through to lactation are lacking [2]. Women’s dietary habits during pregnancy are crucial for maternal and fetal health. During this time, a woman’s energy and nutrient needs increase to support the growth and development of the fetus [3].

A healthy diet during all stages of pregnancy should be rich in vegetables, fruits, legumes, olive oil, nuts, and fish. This type of diet promotes a balanced intake of proteins, high-quality fats (including essential and polyunsaturated fatty acids), and carbohydrates that are high in plant fibers and have a low glycemic index. During pregnancy, micronutrients such as minerals and vitamins—including iron, calcium, zinc, iodine, folic acid, vitamin D, and carotenoids—are often at reduced levels. It is advisable to systematically and carefully administer nutritional supplements to support both pregnancy and breastfeeding processes [4,5,6]. Numerous studies have examined pregnant women’s nutritional profiles and dietary habits, employing various assessment tools such as questionnaires, personal diaries, and nutritional plans. The Food Frequency Questionnaire (FFQ) is an increasingly utilized assessment tool among this population. It has recently been validated within the Greek pregnant population and provides a reliable assessment of nutrient intake based on dietary habits in Greece [7].

Furthermore, Bioelectrical Impedance Analysis (BIA) is a convenient, non-invasive, well-tolerated, and reliable method that offers a quick clinical assessment of body composition and hydration status in pregnant and postpartum women. During pregnancy, significant changes in body composition occur across the trimesters to support fetal growth and maternal adaptation. The Body Fat Mass Index (BFMI) progressively increases, particularly in the second trimester, as fat stores accumulate to meet energy demands in late pregnancy and lactation [8]. To and Wong [9] showed that body fat composition (%) was statistically lower early in pregnancy (<20 weeks) compared to the late third trimester (36–38 weeks) (31.6 ± 5.7 vs. 38.5 ± 5.1, *p* < 0.001). Conversely, the fat-free mass index (FFMI) also rises due to increases in total body water (TBW), which is distributed between extracellular water (ECW) and intracellular water (ICW). The nonfat tissues gained during pregnancy (such as edema fluid, the fetus, amniotic fluid, and plasma) contain a much higher water content. During pregnancy, the water content in lean tissue can rise from about 72.5% at 10 weeks to roughly 75.0% at 40 weeks, with pronounced ECW expansion, especially in women with generalized edema. Interestingly, previous research has revealed that pregnant women experiencing generalized edema retain over 3 kg (6.6 lb) of ECW compared to those without edema or those with edema limited to the legs [10]. This increase can result in a substantial underestimation of body fat, potentially by 50% or more, for those gaining 3 to 4 kg of fat [11].

Additionally, Body Cell Mass (BCM), representing metabolically active tissues, increases gradually during pregnancy. This growth is essential for accommodating the developing fetus and supporting maternal tissues. The expansion of BCM contributes to the overall rise in the Basal Metabolic Rate (BMR) observed during gestation [12]. Butte et al. [13] showed that the BMR increases gradually during pregnancy trimesters, with a mean rate of 10.7 ± 5.4 kcal per gestational week. However, the ratio of BMR to body weight (BMR/BW) typically decreases in the later stages of pregnancy, reflecting the disproportionate gain in water and fat relative to metabolic tissue [14]. Despite its limitation that BIA cannot differentiate between the fetal and maternal tissues [15], studies have shown that BIA has been used and correlated with different medical conditions during pregnancy, such as excessive gestational weight gain, preeclampsia, gestational diabetes mellitus, and hypertension [16,17,18,19,20]. Research also indicates that BIA offers greater predictive value for gestational and postpartum outcomes than body mass index (BMI), and thus, it should be considered in relevant studies [18,21].

In addition, pregnant women are advised to engage in moderate-intensity exercise for at least 30 min on most days of the week, based on the American College of Obstetricians and Gynecologists (ACOG) recommendations. Staying physically active during pregnancy, having a normal body weight, and following a healthy diet can positively impact various aspects of pregnancy and overall well-being [22]. However, despite any benefits of physical activity, it is concerning that pregnant women spend more than 50% of their time displaying sedentary behavior [23,24]. Until recently, no studies have measured physical activity levels and FFQ in Greek pregnant women. Additionally, research examining the relationships among maternal dietary intake, physical activity levels, and body composition during pregnancy is limited, with no studies conducted in Greece. This study aimed to assess maternal dietary intake, physical activity status, and body composition during pregnancy in the Greek population, as well as to examine the possible correlations between the above factors.

## 2. Materials and Methods

### 2.1. Recruitment

Pregnant women were recruited from private and public gynecologic clinics of the Prefecture of the Western Macedonia, Ptolemaida, Greece. Inclusion criteria were defined as follows: (a) age greater than or equal to 18 years, (b) gestational age confirmed by early ultrasound, and (c) compliance with the typical examination by the supervising gynecologist. In contrast, exclusion criteria included: (a) age < 18 years, (b) history of previously confirmed serious illness that may impact maternal and/or fetal tissues, (c) twin pregnancy, (d) receiving medication that may impact BIA analysis, and (e) failure to adhere to the monthly assessment of pregnancy outcomes by the same gynecologist, which may result in missing gestational data.

### 2.2. Sample Size Calculation

By using a one-way ANOVA test of significance (with the significance level at *p* < 0.05) to achieve a power of 80%, we found that a total of 44 participants were required. To be more precise, for *n* = 44, the power was 0.824, assuming there was a 5% chance of making a type I error and a 17.6% chance of making a type II error, considering that the power was equal to 1-beta (1-beta ⇔ 1-0.824). Our study enrolled a total of 49 participants. Moreover, the group size allocation for the overall test was as follows: *n* = 10 for the first trimester, *n* = 19 for the second trimester, and *n* = 15 for the third trimester. In our study, we recruited and evaluated a total of 49 pregnant women who were assigned into 3 non-equal groups based on their pregnancy trimester (first trimester: *n* = 11, second trimester: *n* = 22, third trimester: *n* = 16).

### 2.3. Study Design

Initially, the detailed research protocol was submitted and approved by the University of Western Macedonia Research Ethics Committee and received relevant approval (protocol number: 31/2023). Subsequently, an invitation to participate in the study was announced to pregnant women and residents of the Kozani Prefecture. The invitation provided a detailed description of the study’s purpose, significance, and methodology. Women who volunteered to participate in the study were asked to give written informed consent to the study, which followed the ethical principles of the Declaration of Helsinki (2013). Then they were asked to fill in the Greek version of the Pregnancy Physical Activity Questionnaire (PPAQ), the validated Food Frequency Questionnaire (FFQ) for pregnant women, and to undergo a Bioelectrical Analysis (BIA) for the evaluation of their body composition.

### 2.4. Bioelectrical Impedance Analysis (BIA)

Body composition indices were estimated using bioelectrical impedance analysis (BIA) with a Quadscan 4000 machine (Bodystat, Warwickshire, UK). To ensure accurate BIA measurements, women were advised to avoid eating or drinking for at least 2 h before the test. They were also instructed to refrain from exercise or physical activity for at least 12 h before the test and to inform the researcher about the possible administration of any medication due to an emergency within the time of the test, potentially affecting BIA results. Fortunately, none of the participants reported taking any drugs in the 12 h preceding the BIA assessment. The indices that were analyzed by using two pairs of disposable electrodes in women’s upper and lower limbs were body fat (BF), the body fat mass index (BFMI), the fat-free mass index (FFMI), body cell mass (BCM), the basal metabolic rate (BMR), the ratio of basal metabolic rate and body weight (BMR/BW), extracellular water (ECW), intracellular water (ICW), total body water (TBW), and the phase angle (PhA).

### 2.5. Food Frequency Questionnaire (FFQ)

The present study used the FFQ designed and validated for Greek pregnant women’s dietary habits by Apostolopoulou et al. [7]. The FFQ consists of 46 foods and drinks (categorized into 14 food groups) that reflect Greek nutritional habits and their consumption frequency. For each question, standard portion sizes were explained by weight (grams) or volume (milliliters). These explanations used simple visual representations, such as slices, cups, and tablespoons, or comparisons to the size of a fist or palm. Consumption frequency was noted using four different options: never or rarely, times per day, times per week, and times per month. The reported frequencies were subsequently converted into daily servings. For the dietary data results extraction, data were first gathered in a workbook (Excel 11, Microsoft, Washington, DC, USA). The nutrient composition of food items was obtained from reputable sources, including the Food Composition Tables and the Composition of Greek Cooked Food and Dishes, to ensure the accuracy and reliability of our findings [25]. The portion sizes in the database were modified according to suggestions from the Greek-validated FFQ [7]. To calculate dietary intake, the frequency of the reported weekly or monthly food consumption was converted into a daily figure by dividing that intake by the average frequency. The total energy and macronutrient values were obtained using the nutrition software Nutri Survey (http://www.nutrisurvey.de).

### 2.6. Pregnancy Physical Activity Questionnaire (PPAQ)

The PPAQ is a self-administered questionnaire for pregnant women that takes about 10 min to complete. It includes questions on how much time participants spend on 32 daily activities. The questions are categorized as follows:

- household/caregiving activities (13 questions)
- occupational activities (5 questions)
- sports/exercise (8 questions)
- transportation (3 questions)
- inactivity (3 questions)

Participants were asked to select the category that best reflected the time spent on each activity daily or weekly during their current gestational trimester. For each question, the duration ranged from 0 to 6 or more hours per day and from 0 to 3 or more hours per week. At the end of the PPAQ, an open-ended section allowed respondents to list activities that were not mentioned. Sleeping was not included [26,27]. For the PPAQ energy expenditure calculation, the time spent on each activity was multiplied by its intensity, resulting in a measurement of weekly energy expenditure expressed in the specific metabolic equivalent (MET) hours per week (MET.h.wk-1) for each activity. Each activity was assigned an MET to assess its intensity [sedentary (less than 1.5 METs), light (between 1.6 and 2.9 METs), moderate (between 3.0 and 5.9 METs), and vigorous (greater than 6.0 METs)], according to the “Compendium of Physical Activities: An Update of Activity Codes and MET Intensity Levels” [26,27,28,29]. The formulas used to calculate the total weekly energy expenditure and that related to different intensities and types of physical activity were based on Santos et al. [30]. The Greek version of the PPAQ, which was translated and adapted based on Greek culture, was used [10].

### 2.7. Human Research Protection Data

Participants were clearly informed through a written consent form that their participation in the study would be anonymous. Personal data assurance and protection were emphasized for all participants in compliance with GDPR (EU Regulation 2016/679).

### 2.8. Statistical Analysis

Statistical analysis used IBM SPSS Statistics for Windows (Version 29.0, IBM Corp., 2020, Armonk, NY, USA). The Kolmogorov–Smirnov test assessed normal distribution. Normally distributed variables are presented as mean ± standard deviation, while non-parametric variables are reported as median (interquartile range; 25th–75th percentiles) and range [min, max]. The number of subjects and their corresponding percentage values (%) were provided with the qualitative data. A one-way ANOVA was performed to assess the impact of the pregnancy trimester on BIA indices. Pearson correlation analyzed the relationship between estimated energy and nutrients from the FFQ and pregnancy trimester. Multiple linear regression was used to assess the impact of confounding factors—including age, BMI, employment status, and BFMI—on PPAQ results. Lower PPAQ levels observed during the gestational period may be influenced by increases in BMI, BFMI, and FFMI during pregnancy trimesters, or they may be a consequence of unemployment status often seen in the third trimester of pregnancy. The significance level for determining statistical differences was set at *p* < 0.05.

## 3. Results

### 3.1. Participant Characteristics

Table 1 shows the demographic and anthropometric characteristics of the pregnant women. Forty-nine pregnant women, with a mean age of 31.08 ± 4.90 years old and a BMI 28.00 ± 4.61 kg/cm^2^, who met the inclusion criteria, participated in the study.

### 3.2. Bioelectrical Impedance Analysis Results

Table 2 presents the BIA results in pregnant women. Additionally, the analysis of variance showed that the effect of the pregnancy trimester on BF (F(2,46) = [14.824], *p* < 0.001), BFMI (F(2,46) = [7.106], *p* < 0.001), FFMI (F(2,46) = [9.142], *p* = 0.003), BCM (F(2,46) = [7.113], *p* = 0.002), ECW (F(2,46) = [10.215], *p* < 0.001), ICW (F(2,46) = [18.478], *p* < 0.001), and LEAN (F(2,46) = [6.973], *p* < 0.001) was statistically significant (Table 3 and Table 4).

### 3.3. FFQ Results

**Figure 1:**
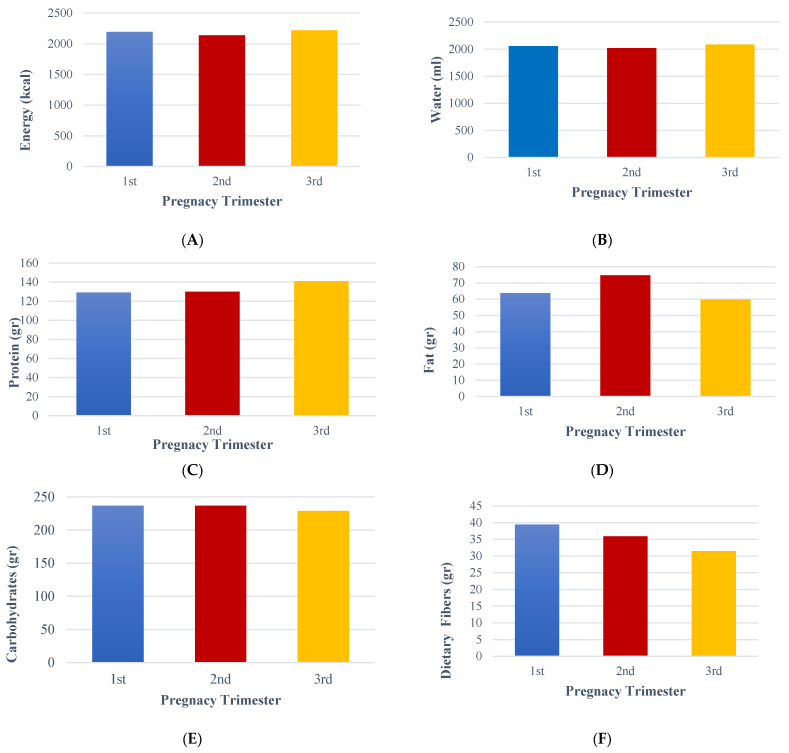
Comparison of the estimated energy and nutrients based on the FFQ per pregnancy trimester. The (**A**) subfigure shows the mean energy consumption (kcal) per pregnancy trimester. The (**B**) subfigure represents the average water consumption (lt) per pregnancy trimester. The (**C**) subfigure represents the average protein consumption (gr) per pregnancy trimester. The (**D**) subfigure shows the mean fat consumption (gr) per pregnancy trimester. The (**E**) subfigure shows the average carbohydrates consumption (gr) per pregnancy trimester. The (**F**) subfigure shows the average dietary fibers consumption (gr) per pregnancy trimester.

Table 5 presents the distribution of six macronutrient groups assessed using the FFQ. The results indicate that pregnant women consume a median of 2135 kcal and 2012.10 mL of water per day, regardless of their trimester. Pearson correlation analysis revealed a significant positive correlation between pregnancy trimester and energy intake (r = 0.795, *p* < 0.001), water intake (r = 0.759, *p* < 0.001), fat intake (r = 0.535, *p* = 0.029), and dietary fiber intake (r = 0.310, *p* < 0.001) (Table 6). Additionally, Pearson correlation analysis showed a significant positive correlation between economic status and both energy intake (r = 0.389, *p* = 0.028) and protein intake (r = 0.410, *p* = 0.020), while a negative correlation was observed between economic status and carbohydrate intake (r = −0.354, *p* = 0.047) (Table 7). These significant correlations underscore the crucial role of these factors in the context of pregnancy. Figure 1 and Table 8 present the distribution of the six groups of macronutrients during the three pregnancy trimesters.

### 3.4. PPAQ Results

**Figure 2:**
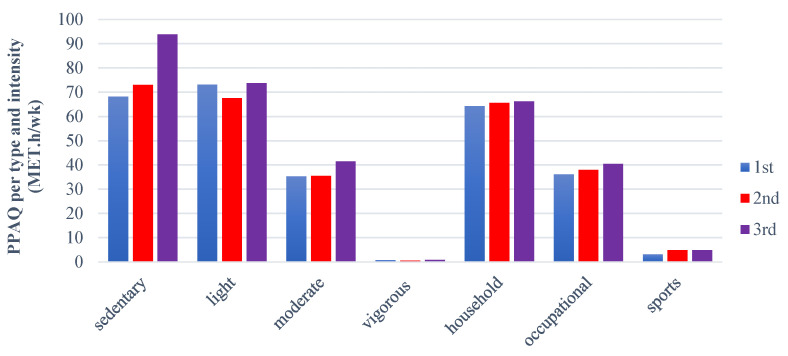
Comparison of the type and intensity of PA, deducted from the PPAQ per pregnancy trimester.

According to the type and intensity of physical activity, deducted from the PPAQ in women categorized based on their pregnancy trimester, the results showed that women in the third trimester are statistically more sedentary than those in the second and first trimesters, respectively. This finding underscores the need for interventions to promote physical activity in the third trimester. In addition, it was observed that pregnant women had similar light, moderate, and vigorous levels of physical activity, as well as that they spent almost equally their physical activity in household/caregiving and occupational jobs (Table 9 and Figure 2). Lastly, multiple regression analysis revealed the relationship between the total PPAQ score and various independent variables (such as age, BMI, and pregnancy trimester). The results showed that pregnancy trimester (*p* = 0.005), employment status (*p* = 0.040), economic status (*p* = 0.037), and higher BMI (*p* = 0.013), BFMI (*p* = 0.017), and FFMI (*p* = 0.024) values made a significant contribution to the model (Table 10). To be more precise, the results revealed that 64.4% of the variability observed in the total PPAQ score was explained by the regression model (R^2^ = 0.644, F = 5.206, *p* < 0.001).

## 4. Discussion

Our study found that the trimester of pregnancy significantly affects various indices of BIA and nutrient intake, as assessed by the FFQ. Additionally, physical activity levels decline considerably during pregnancy, and most pregnant women do not meet the recommended activity levels set by the ACOG. More precisely, regarding the body composition measurement, pregnant women during the third trimester had, on average, a slight but statistically significant increase in BF, BFMI, ECW, ICW, and LEAN compared to those in the first and second trimesters. It is notably accepted that weight gained during pregnancy is associated with fat mass rather than free-fat mass, and these factors, measured by BIA, have been considered better maternal nutritional status predictors than BMI [15,31], even though the differentiation between maternal and fetal fat and free-fat mass is not possible [32], and the abnormal fluid distribution during pregnancy makes different impedance methods, such as BIA and air displacement plethysmography (ADP), in need of further validation [33]. In contrast to these references, especially in high-income countries, BIA is considered a valid method for evaluating TBW and ECW increases from the first stages of pregnancy until delivery [34,35]. Bai et al. [36] showed that BIA’s test-retest repeatability was excellent and consistent during all three trimesters of pregnancy. Their study also showed that BMI, TBW, and fat mass differed significantly between pregnancy trimesters. However, other studies have revealed that BIA is a valuable tool for predicting preeclampsia, mainly by measuring the ECW/ICW ratio before the clinical onset of the disease [37,38,39]. Trindade et al. [40], using the BIA method, observed that pregnant women with lower ECW/ICW and ECW/TBW ratios at 17–20 weeks of gestation were more likely to develop preeclampsia. These findings highlight the significance of increased ICW over increased ECW in predicting this condition. Furthermore, the significant increases in TBW, ECW, BCM, free fat mass, and fat mass during the trimesters have been identified as risk factors that substantially raise the likelihood of developing gestational diabetes mellitus [41]. While research indicates that maternal fat mass changes can range from a decrease of 10 kg to an increase of 15 kg by the end of pregnancy [42]. These changes show a positive correlation with gestational weight gain [19].

Furthermore, our study found that BCM (*p* = 0.002) was significantly higher in women during the third trimester of pregnancy compared to those in the first and second trimesters. However, the BMR and the BMR/BW ratio did not show any significant differences across the trimesters of pregnancy. The small sample size may explain this, leading to a slight increase in BCM. BCM, representing the metabolically active components of the body, increases during pregnancy. This growth is essential for accommodating the developing fetus and supporting maternal tissues. The expansion of BCM contributes to the overall rise in BMR observed during gestation. Lof et al. [14], to identify factors associated with variability in the BMR response to pregnancy, noticed that, during the 14th week of pregnancy, BMR was statistically correlated with both weight gain and pre-gestational total body fat percentage. While during the 32nd week of gestation, high BMR was significantly associated with FFM, body weight, cardiac output, free triiodothyronine, and total body fat [14]. Based on the abovementioned body composition analysis with BIA or other related methods during pregnancy, trimesters may contribute to significant clinical considerations.

Moreover, our study, based on the FFQ results, unveiled a significant positive correlation between energy, water, fat, and dietary fibers with pregnancy trimester. More precisely, it revealed that pregnant women tend to consume higher amounts of energy and fat during the second trimester. In contrast, they consume less water during the first and second trimesters. Additionally, during the third trimester, there is a decrease in the intake of dietary fiber and carbohydrates, while the consumption of protein increases compared to the other trimesters. FFQ is particularly important during pregnancy as it assesses food consumption in this group. It generally demonstrates good methodological quality and adequate correlation coefficients compared to reference methods [43]. The FFQ has been validated in numerous countries, each with different dietary habits [43]. In a study by Brito et al. [44], which aimed to validate the FFQ in pregnant women in Brazil, researchers found that the validation and calibration of the FFQ increased the accuracy of the instrument when compared to the reference standard (24hR) and concluded that this instrument can effectively be used to examine the association between maternal feeding during pregnancy and maternal and fetal health. Similar results were found from the validation of FFQ in Finnish [45], South Eastern Spanish [46], and Caucasian women [47]. In a study conducted by Chandonnet et al. [48], in China, the average nutrient intakes measured using an FFQ were comparable to those previously reported in the Chinese National Nutrition and Health Survey from 2002, specifically among women living in rural areas. However, there were notably low protein, fat, iron, and zinc intakes. Among the participants, 54% were at risk of inadequate energy intake. Furthermore, many pregnant women were found to have insufficient intakes of folate (97%) and zinc (91%). Our study showed that the average energy and water intake were 2135 kcal and 2012.10 mL of water per day, respectively, regardless of their trimester. In agreement, Apostolopoulou et al. [7], who recently validated the FFQ based on the culture and dietary habits of Greek pregnant women, observed similar energy (1933 kcal) and water (2063 mL) intake results using the FFQ in Greek pregnant women. However, there is a significant gap in our understanding of the dietary behavior and nutritional status of pregnant women in Greece. This study, therefore, aimed to fill this gap and shed light on the food consumption patterns during pregnancy in Greek pregnant women, underlining the necessity and potential impact of this research.

Additionally, our study unveiled significant correlations between economic status and estimated energy and nutrients, such as protein and carbohydrates, based on the FFQ. Several studies have shown that economic status, often measured by annual income, is significantly correlated with dietary intake and food choices among pregnant women, as assessed through the FFQ. Higher-income women tend to have greater access to a diverse and nutrient-rich diet, including higher consumption of protein, fruits, vegetables, and healthy fats, while lower-income women may have limited access to fresh and nutrient-dense foods, often leading to the higher consumption of processed and energy-dense options. Pearson correlation analyses in various studies have demonstrated positive associations between economic status and the intake of total energy, protein, and micronutrient-rich foods, while negative correlations have been observed with carbohydrate intake, particularly from refined sources. Recent studies, such as the study by Ficadu et al. [49], have demonstrated that socioeconomic status significantly influences dietary patterns and nutritional status among pregnant women. While Alemu et al. [50] found that factors such as place of residence, employment status, average monthly household income, the frequency of meals consumed each day, and receiving dietary counseling during antenatal care strongly predicted 55.4% of dietary diversity. These disparities highlight the influence of financial resources on maternal nutrition, which can impact pregnancy outcomes and fetal development.

Furthermore, regarding the physical activity status of pregnant women, the PPAQ is considered an easy, self-administered questionnaire that measures and assesses the levels of physical activity in pregnant women, providing significant results [19]. PPAQ has been validated and translated into many languages, including Greek [1]. Our study showed that pregnant women are statistically more sedentary during the third trimester. The results also indicated that their levels of light activity remained similar during both the first and third trimesters, while moderate activity, household, occupational, and sports levels were higher in the third trimester, although these differences were not statistically significant. Papazian et al. [46] observed that 51% and 1.7% of women engage in light and high-intensity physical activity during pregnancy, respectively, while 18% have a sedentary lifestyle. They also observed that women during their second trimester were more active in terms of household tasks and caregiving, whereas women in their third trimester were physically more active in occupational activities. Borodulin et al. [43], in a previous study that enrolled 1482 pregnant women, found that their overall physical activity levels decreased during pregnancy, particularly in caregiving, outdoor household tasks, and recreational activities. They also observed that women who remained active during the second and third trimesters of pregnancy reported higher levels of physical activity across all types of exercise compared to those who either became active or inactive during their pregnancy. Despite this, most of these women did not meet the recommended levels of physical activity according to the ACOG guidelines.

Interestingly, in a systematic review by Meander et al. [44], it was found that physical activity during pregnancy was linked to lower average gestational weight gain and a reduced risk of emergency cesarean sections. Additionally, a more sedentary lifestyle during pregnancy was weakly associated with increased blood loss during labor and postpartum. Both physical activity and a sedentary lifestyle were significantly related to self-reported health during pregnancy. In the studies analyzed, only 27.3% of participants achieved the recommended physical activity level by weeks 32 to 34 of their pregnancy. This is lower than a previous study conducted in Sweden, which found that 47% of pregnant women reached the recommended physical activity level by 10 weeks gestation [45]. One possible explanation for the differing results between studies is that pregnant women often reduce their physical activity levels as pregnancy progresses. Additionally, varying methods used to assess participants’ physical activity may have influenced these results [46].

Our study also found that the total PPAQ score was significantly correlated with BMI, pregnancy trimester, BFMI, and FFMI. Unlike our findings, Papazian et al. [47] found that maternal age did not correlate with the total score of the PPAQ among the participants. However, self-reported nutritional and physical status showed a significant correlation with levels of physical activity. Despite these conclusions, the fact that they observed increased occupational and household activity levels during the last trimester highlighted that working mothers stay active until the final due date to accomplish family and work duties. In agreement with these facts, our study unveiled a significant correlation between employment status and PPAQ levels. In addition, total PPAQ levels were significantly correlated with BFMI and FFMI. As noted earlier, fat mass increases during pregnancy and is associated with weight gain [15,39]. In this context, lower physical activity levels contribute to increased fat mass, leading to a higher BMI by the end of the pregnancy. Sedentary behavior is common among pregnant women, which can result in increased gestational weight gain and decreased physical activity levels. Chandonnet et al. [48], found that obese pregnant women are active mainly in household and caregiving activities, with a low total PPAQ score, while Santos et al. [49], reported that women allocated most of their weekly time to domestic, occupational, and leisure activities, excluding sports.

In summary, this study has both strengths and limitations. Firstly, it assessed Greek pregnant women’s dietary intake, body composition, and physical activity levels. Secondly, significant correlations were found between the pregnancy trimester and the indices of the FFQ, as well as between the PPAQ and factors such as age, BMI, FFMI, BFMI, employment, and economic status. Finally, it was noted that body composition, FFQ results, and physical activity levels vary across different pregnancy trimesters. The study also has several limitations that should be considered. First, the small number of participants may limit the generalizability of the findings, as a larger and more diverse sample could provide more robust results. Second, using an FFQ introduces potential biases, such as underreporting and overreporting of dietary intake, which could affect the accuracy of the data. These biases may arise due to participants’ recall errors or social desirability bias. Future studies could incorporate additional dietary assessment methods, such as 24-h dietary recalls or biomarkers, to mitigate this and validate FFQ data. Lastly, the study did not evaluate other dietary assessment tools or body composition measurement methods for pregnant women, which limits the ability to compare the FFQ’s effectiveness with alternative approaches. Future research could address this by including multiple dietary assessment methods and body composition analyses to enhance the reliability of the findings.

## 5. Conclusions

In summary, pregnancy trimesters significantly influence various BIA indices and nutrient intake as assessed by the FFQ, while physical activity levels decline sharply throughout pregnancy. Future studies should track the dietary intake, body composition, and physical activity levels of the same women across all trimesters to provide a more comprehensive understanding of these changes. Additionally, further research is needed to explore how these factors impact pregnancy outcomes and newborn characteristics.

## Figures and Tables

**Table 1:** Clinical characteristics of pregnant women.

Variable	Values
Age (years)	31.08 ± 4.90
Height (cm)	168.33 ± 5.12
Weight (kg)	73.20 ± 11.80
BMI (kg/cm^2^)	28.0 ± 4.61
Marital status	
Single	3 (6.12%)
In a relationship	17 (34.69%)
Married	20 (40.81%)
Divorced	9 (18.36%)
Education	
Primary education	4 (8.16%)
Secondary education	28 (57.14%)
Higher education	17 (34.69%)
No education	-
Employment status	
Unemployed	13 (26.53%)
Employed	36 (73.46%)
Economical status (annual income)	
Very low (up to 5000 euros)	4 (8.16%)
Low (ranging from 5001 to 10,000 euros)	8 (16.32%)
Median (ranging from 10,001 to 20,000 euros)	25 (51.02%)
High (ranging from 20,001 to 30,000 euros)	10 (20.40%)
Very high (over 30,000 euros)	2 (4.08%)
Medical history	
Anemia	7 (14.28%)
Drug medication	
Pregnancy vitamins and/or minerals	49 (100.00%)

Note: Data are expressed as mean ± SD and as *n* (%). BMI: Body Mass Index.

**Table 2:** Bioelectrical impedance analysis in pregnant women.

Variable	Values
BF (kg)	25.59 ± 7.31
BF (%)	35.50 ± 5.85
BFMI (kg/m^2^)	9.56 ± 2.87
FFMI (kg/m^2^)	17.06 ± 1.47
BCM (kg)	25.14 ± 3.41
BMR (kcal)	1534.37 ± 152.55
BMR/BW (kcal/kg)	21.48 ± 2.46
ECW (lt)	15.61 ± 1.79
ECW (%)	21.99 ± 1.90
ICW (lt)	17.76 ± 2.71
ICW (%)	24.43 ± 1.80
TBW (lt)	33.77 ± 8.30
TBW (%)	45.68 ± 4.05
LEAN (%)	64.84 ± 6.04
LEAN (kg)	46.21 ± 6.26
DRY LEAN (kg)	13.77 ± 2.93
PhA (°)	5.41 ± 0.50

Note: Data are expressed as mean ± SD. BF: body fat; BFMI: body fat mass index; FFMI: fat-free mass index; BCM: body cell mass; BMR: basal metabolic rate; BMR/BW: ratio of basal metabolic rate and body weight; ECW: extracellular water; ICW: intracellular water; TBW: total body water; PhA: phase angle.

**Table 3:** Results of the one-way ANOVA analysis with the pregnancy trimester as the differentiating variable and each of the BIA indices as dependent variables.

Variable	F	df (Between Groups, Within Groups)	*p*-Value	pη^2^
BF(kg)	14.824	2.46	*p* < 0.001 *	0.890
BFMI (kg/m^2^)	7.106	2.46	*p* < 0.001 *	0.634
FFMI (kg/m^2^)	9.142	2.46	*p* = 0.003 *	0.685
BCM (kg)	7.113	2.46	*p* = 0.002 *	0.690
BMR (kcal)	1.994	2.46	*p* = 0.110	0.224
BMR/BW (kcal/kg)	4.002	2.46	*p* = 0.062	0.323
ECW (lt)	10.215	2.46	*p* < 0.001 *	0.756
ICW (lt)	18.478	2.46	*p* < 0.001 *	0.987
TBW (lt)	2.359	2.46	*p* = 0.125	0.189
LEAN (kg)	6.973	2.46	*p* < 0.001 *	0.624
DRY LEAN (kg)	1.980	2.46	*p* = 0.172	0.187

Note: Data are expressed as F-value, degrees of freedom (df) between groups/within groups, level of statistical significance (*p*-value) and partial eta squared (pη^2^). BF: body fat; BFMI: body fat mass index; FFMI: fat-free mass index; BCM: body cell mass; BMR: basal metabolic rate; BMR/BW: ratio of basal metabolic rate and body weight; ECW: extracellular water; ICW: intracellular water; TBW: total body water. * *p* < 0.05.

**Table 4:** Estimated BIA indices in women categorized depending on their trimester.

	1st Trimester (*n* = 11)	2nd Trimester (*n* = 22)	3rd Trimester (*n* = 16)	*p*-Value
BF(kg)	26.76 ± 8.29	25.99 ± 8.97	24.06 ± 4.60	*p* < 0.001 *
BFMI (kg/m^2^)	11.33 ± 3.11	9.28 ± 3.32	8.90 ± 1.65	*p* < 0.001 *
FFMI (kg/m^2^)	17.11 ± 1.60	16.65 ± 1.48	17.81 ± 1.42	*p* = 0.003 *
BCM (kg)	23.55 ± 2.82	25.35 ± 3.24	25.82 ± 2.68	*p* = 0.002 *
ECW (lt)	14.50 ± 1.49	15.64 ± 1.35	16.31 ± 1.81	*p* < 0.001 *
ICW (lt)	16.48 ± 1.98	18.06 ± 2.87	18.06 ± 1.86	*p* < 0.001 *
LEAN (kg)	41.73 ± 4.02	43.99 ± 4.31	48.26 ± 5.94	*p* < 0.001 *

Note: Values are presented as mean ± standard deviation for variables with a normal parametric distribution. BF: body fat; BFMI: body fat mass index; FFMI: fat-free mass index; BCM: body cell mass; BMR: basal metabolic rate; BMR/BW: ratio of basal metabolic rate and body weight; ECW: extracellular water; ICW: intracellular water; TBW: total body water. * *p* < 0.05.

**Table 5:** Median and quartiles (Q1 and Q3) of the estimated energy and nutrients based on the FFQ in pregnancy.

Nutrients	FFQ
Energy (kcal)	2135.00 (199.48), [1875.10, 2600.00]
Water (mL)	2012.10 (189.43), [1786.20, 2405.30]
Protein (g)	131.60 (15.55), [90.60, 153.70]
Fat (g)	65.00 (14.25), [43.00, 110.00]
Carbohydrates (g)	241.50 (71.17), [169.00, 299.00]
Dietary fibers (g)	33.50 (19.25), [20.00, 58.00]

Note: Values are presented as median (interquartile range), [min, max] for variables with a non-parametric distribution. FFQ: Food Frequency Questionnaire.

**Table 6:** Pearson correlations between the estimated energy and nutrients based on the FFQ and pregnancy trimester.

Nutrients	Pearson	*p*-Value
Energy (kcal)	0.795	*p* < 0.001 *
Water (mL)	0.759	*p* < 0.001 *
Protein (g)	0.211	*p* = 0.147
Fat (g)	0.535	*p* = 0.029 *
Carbohydrates (g)	0.111	*p* = 0.272
Dietary fibers (g)	0.310	*p* < 0.001 *

Note: * *p* < 0.05.

**Table 7:** Pearson correlations between the estimated energy and nutrients based on the FFQ and economic status.

Nutrients	Pearson	*p*-Value
Energy (kcal)	0.389	*p* = 0.028 *
Water (mL)	−0.008	*p* = 0.965
Protein (g)	0.410	*p* = 0.020 *
Fat (g)	−0.152	*p* = 0.407
Carbohydrates (g)	−0.354	*p* = 0.047 *
Dietary fibers (g)	0.099	*p* = 0.591

Note: * *p* < 0.05.

**Table 8:** Estimated energy and nutrients based on the FFQ in women categorized depending on their trimester.

	1st Trimester (*n* = 11)	2nd Trimester (*n* = 22)	3rd Trimester (*n* = 16)	*p*-Value
Energy (kcal)	2240.07 (147.79), [1875.10, 2600.00]	2140.24 (167.62), [1910.00, 2278.30]	2220.23 (141.81), [2075.11, 2390.00]	*p* < 0.001 *
Water (mL)	2059.37 (164.71), [1786.20,2405.31]	2022.97 (142.87), [1789.30, 2189.42]	2089.48(112.26), [1957.90, 2211.81]	*p* < 0.001 *
Protein (g)	129.17 (19.43), [90.60, 153.00]	129.92 (12.51), [100.60, 153.70]	141.18 (10.00), [132.00, 153.70]	*p* = 0.147
Fat (g)	63.77 (11.86), [45.00, 70.00]	74.76 (20.79), [43.00, 110.00]	59.83 (6.00), [54.00, 69.10]	*p* = 0.029 *
Carbohydrates (g)	236.76 (47.27), [169.00, 291.00]	236.70 (40.79), [169.00, 287.00]	228.96 (14.78), [176.00, 260.00]	*p* = 0.272
Dietary fibers (g)	39.44 (14.69), [21.00, 57.00]	35.88 (12.08), [20.00, 58.00]	31.50 (4.59), [29.00, 36.00]	*p* < 0.001 *

Note: Values are presented as median (interquartile range), [min, max] for variables with a non-parametric distribution. * *p* < 0.05.

**Table 9:** Type and intensity of PA, deducted from the PPAQ, in women categorized depending on their trimester.

	1st Trimester (*n* = 11)	2nd Trimester (*n* = 22)	3rd Trimester (*n* = 16)	*p*-Value
Total score of PPAQ (MET.h/wk)	177.38 ± 33.55	176.65 ± 33.45	209.95 ± 40.62	*p* < 0.001 *
By intensity				
Sedentary	68.21 ± 7.73	73.04 ± 8.12	93.84 ± 21.35	*p* < 0.001 *
Light	73.11 ± 8.82	65.56 ± 12.89	73.77 ± 8.99	*p* = 0.749
Moderate	35.26 ± 10.00	35.48 ± 8.91	41.47 ± 8.41	*p* = 0.151
Vigorous	0.78 ± 0.15	0.56 ± 0.25	0.85 ± 0.65	*p* = 0.700
By type				
Household/Caregiving	64.31 ± 14.78	65.65 ± 12.46	66.18 ± 9.61	*p* = 0.777
Occupational	36.10 ± 12.74	38.10 ± 6.56	40.41 ± 9.13	*p* = 0.332
Sports/exercise	3.16 ± 1.32	4.81 ± 2.39	4.88 ± 2.02	*p* = 0.163

Note: Data are expressed as mean ± SD. PPAQ: Pregnancy Physical Activity Questionnaire. * *p* < 0.05.

**Table 10:** Multiple regression analysis with total score of PPAQ (MET.h/wk) as a dependent variable.

	Unstandardized Coefficients		Standardized Coefficients	*t*-Test	*p*-Value	95.0% Confidence Interval for B	
	B	Std. Error	Beta			Lower Bound	Upper Bound
(Constant)	266.098	57.955		4.591	*p* < 0.001	146.209	385.987
Age (years)	−1.301	0.794	−0.243	−1.638	*p* = 0.115	−2.836	0.342
BMI (kg/cm^2^)	−168.642	63.831	−31.002	−2.111	*p* = 0.013 *	−237.992	−29.544
Pregnancy trimester	16.687	5.305	0.531	3.146	*p* = 0.005 *	5.713	27.660
Employment status	13.291	5.972	0.423	2.226	*p* = 0.040 *	0.692	25.889
Economic status	12.189	5.513	0.334	2.211	*p* = 0.037 *	0.785	23.593
Education	3.933	3.735	0.190	1.053	*p* = 0.303	−3.793	11.658
BF (kg)	2.317	1.864	0.381	1.416	*p* = 0.117	−0.546	4.552
BFMI (kg/m^2^)	173.451	67.330	23.258	2.576	*p* = 0.017 *	34.167	−312.734
FFMI (kg/m^2^)	157.539	11.241	11.241	2.416	*p* = 0.024 *	22.660	292.417
R^2^ = 0.644, F = 5.206, *p* < 0.001							

Note: PPAQ: Pregnancy Physical Activity Questionnaire; BFMI: body fat mass index; FFMI: fat-free mass index; ΒΜΙ: Body Mass Index. * *p* < 0.05.

## Data Availability

The data presented in this study is available on request from the corresponding author. The data is not publicly available due to ethical restrictions.

## Public Involvement Statement

No public involvement in any aspect of this research.

## Guidelines and Standards Statement

This manuscript was drafted against STROBE for crosssectional study research.
